# Crystal structure of nicotinamide ethyl­ene glycol hemisolvate

**DOI:** 10.1107/S2056989026005219

**Published:** 2026-05-22

**Authors:** Tanapat Chunanant, Peerapon Rapeenun, Natthaphong Lertna, Adrian E. Flood

**Affiliations:** a School of Energy Science and Engineering, Vidyasirimedhi Institute of Science and Technology, Rayong, 21210, Thailand; b Department of Chemical Engineering, Rowan University, Glassboro, NJ 08028, USA; University of Aberdeen, United Kingdom

**Keywords:** crystal structure, solvate, hydrogen bonding, nicotinamide

## Abstract

In the title solvate, C_6_H_6_N_2_O·0.5C_2_H_6_O_2_, the asymmetric unit consists of one nicotinamide mol­ecule and half of an ethyl­ene glycol mol­ecule, which is completed by crystallographic inversion symmetry. The dihedral angle between the acetamide group and the pyridine ring is 21.9 (8)°. In the crystal, the components are linked by N—H⋯O and O—H⋯N hydrogen bonds into (102) sheets and weak offset π–π stacking is also observed. Hirshfeld surface analysis indicates that the most important contacts in the structure are H⋯H (42.9%), O⋯H/H⋯O (26.4%), C⋯H/H⋯C (10.6%) and N⋯H/H⋯N (9.2%).

## Abstract 2

The asymmetric unit of the title solvate consists of one nicotinamide mol­ecule and half of an ethyl­ene glycol mol­ecule, which is completed by crystallographic inversion symmetry. The dihedral angle between the acetamide group and the pyridine ring is 21.9 (8)°. In the crystal, the components are linked by N—H⋯O and O—H⋯N hydrogen bonds into (102) sheets and weak offset π–π stacking is also observed.

## 1. Chemical context

Azeotropic mixtures cannot be separated by conventional distillation since the vapor and liquid compositions of two or more component compounds are identical at the azeotropic condition (Rackley, 2010 ▸). Azeotropic distillation is a common method for separation, but the energy and solvent consumption are very high (Speight, 2020 ▸). Because of the high specificity of a crystalline solid, it is possible to effectively separate mol­ecules by crystallization; however, the solvents commonly found in azeotropes typically have low melting points making direct crystallization less promising. Alternatively, the crystallization of solvates may allow for the separation of azeotropic mixtures at ambient conditions, with simple separation possible if only one of the solvents in the azeotropic mixture can form a solvate with a specific coformer. This may make the separation process more efficient, both lowering energy use, solvent use (compared to azeotropic and extractive distillation), and equipment complexity.

A solvate is defined as a crystalline solid that incorporates one or more solvent mol­ecules into its structure (Maheshwari *et al.*, 2018 ▸). They are formed and stabilized by inter­molecular inter­actions such as hydrogen bonds, aromatic π–π stacking and van der Waals forces. The solvate of nicotinamide (C_6_H_6_N_2_O; NAM) and ethyl­ene glycol (C_2_H_6_O_2_; EG) solvate is composed of one mol­ecule of NAM and a half mol­ecule of EG in the asymmetric unit. Even though the space group and the dimensions of the unit cell have already been determined (Wright & King, 1950 ▸; CSD refcode ZZZFOO), its full three-dimensional structure remains unidentified. This paper reports the crystal structure of the title solvate, C_6_H_6_N_2_O.1/2(C_2_H_6_O_2_) (**I**), which was formed by cooling crystallization.
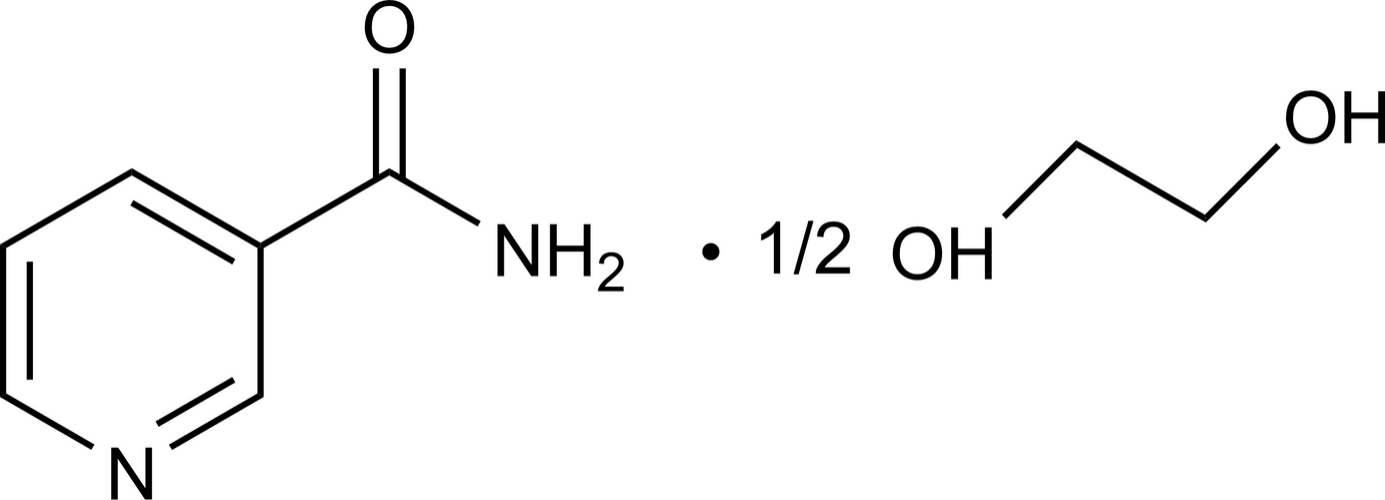


## 2. Structural commentary

**Figure 1:**
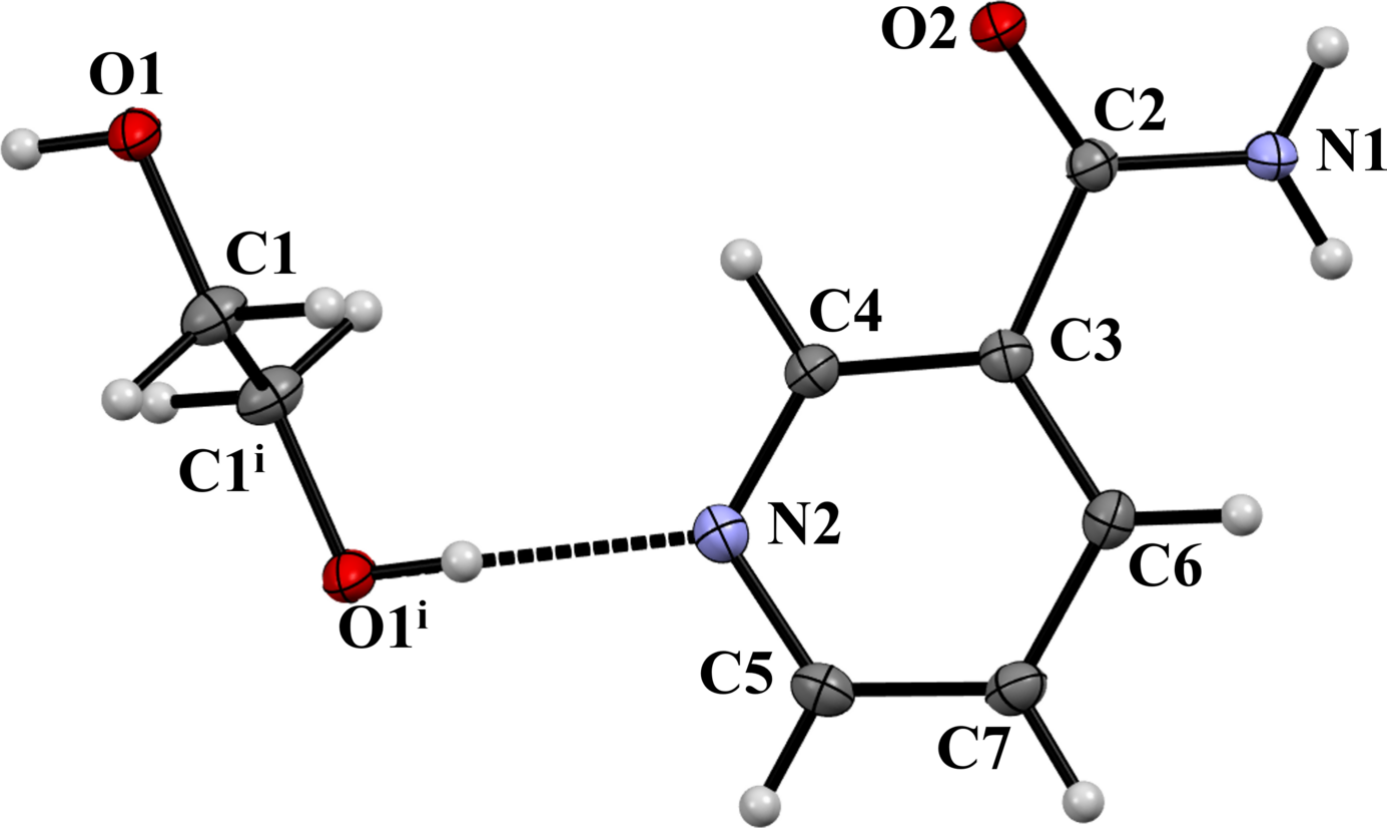
The mol­ecular structure of (**I**) with displacement ellipsoids drawn at the 50% probability level. The hydrogen bond is indicated by a dashed line. Symmetry code: (i) 2 − *x*, 1 − *y*, 1 − *z*.

In agreement with the 1950 study, compound (**I**) crystallizes in the monoclinic *P*2_1_/*c* space group, with one mol­ecule of NAM and a half mol­ecule of EG in the asymmetric unit, as shown in Fig. 1 ▸. The dihedral angle between the C3–C7/N2 aromatic ring and the pendant acetamide group is 21.9 (8)° and the complete EG mol­ecule is generated by inversion symmetry [the centre of symmetry for the asymmetric atoms lies at (1, 1/2, 1/2)]. The C6—C3—C2 and C3—C2—N1 bond angles are 123.35 (9) and 116.94 (9)°, respectively.

## 3. Supra­molecular features

**Figure 2:**
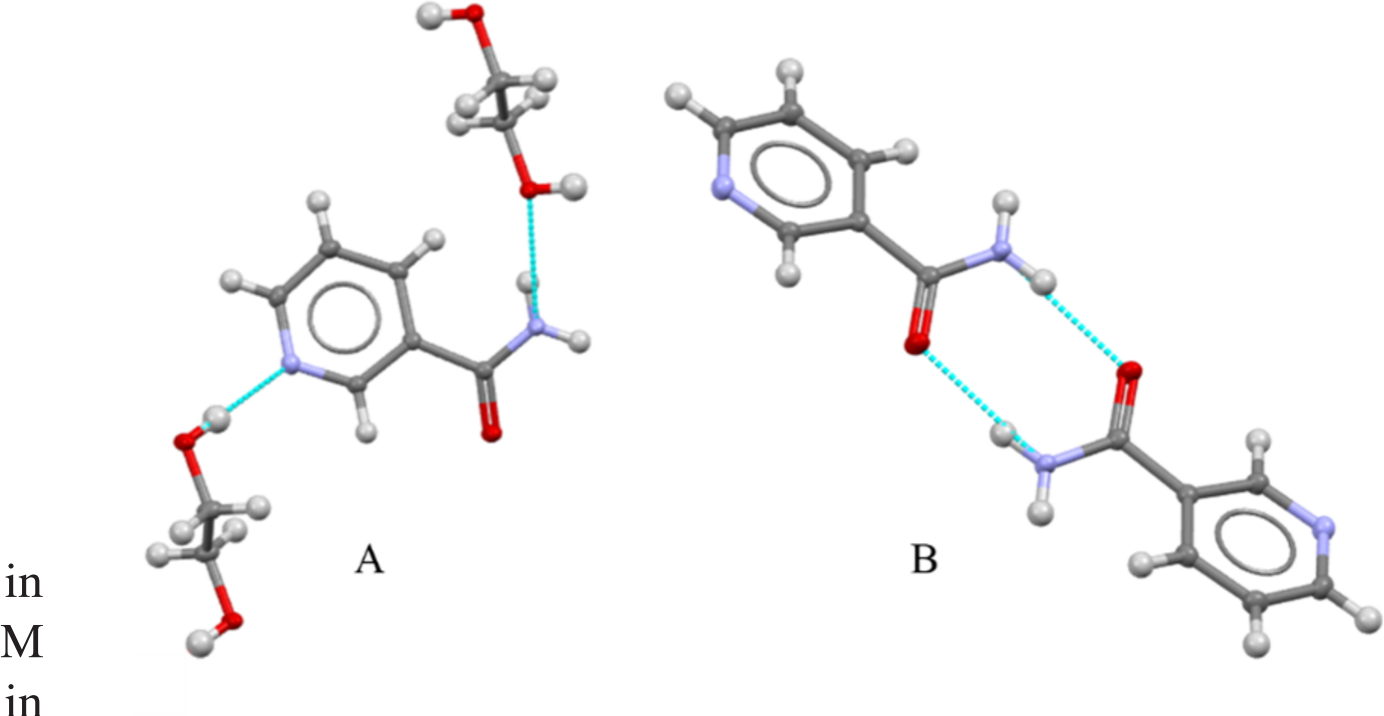
Detail of hydrogen bonds in the crystal structure of (**I**): type A is the inter­action between EG and NAM, including N—H⋯O and O—H⋯N bonds and type B is the pairwise inter­action between two NAM mol­ecules.

In the extended structure, three different types of hydrogen bonds (Table 1 ▸) are the main inter­molecular inter­actions consolidating the structure. The EG oxygen atom (O1) bridges two NAM mol­ecules through two hydrogen-bond inter­actions: it acts as an acceptor for N1—H1*B*⋯O1 and as a donor in O1—H1⋯N2. Additionally, pairwise hydrogen-bonding inter­actions occur between the amide functional groups of neighboring NAM mol­ecules, N1—H1*A*⋯O2, which generate centrosymmetric 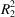
(8) loops. These can be seen in Fig. 2 ▸. Collectively, the hydrogen bonds generate infinite (10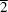) sheets.

**Figure 3:**
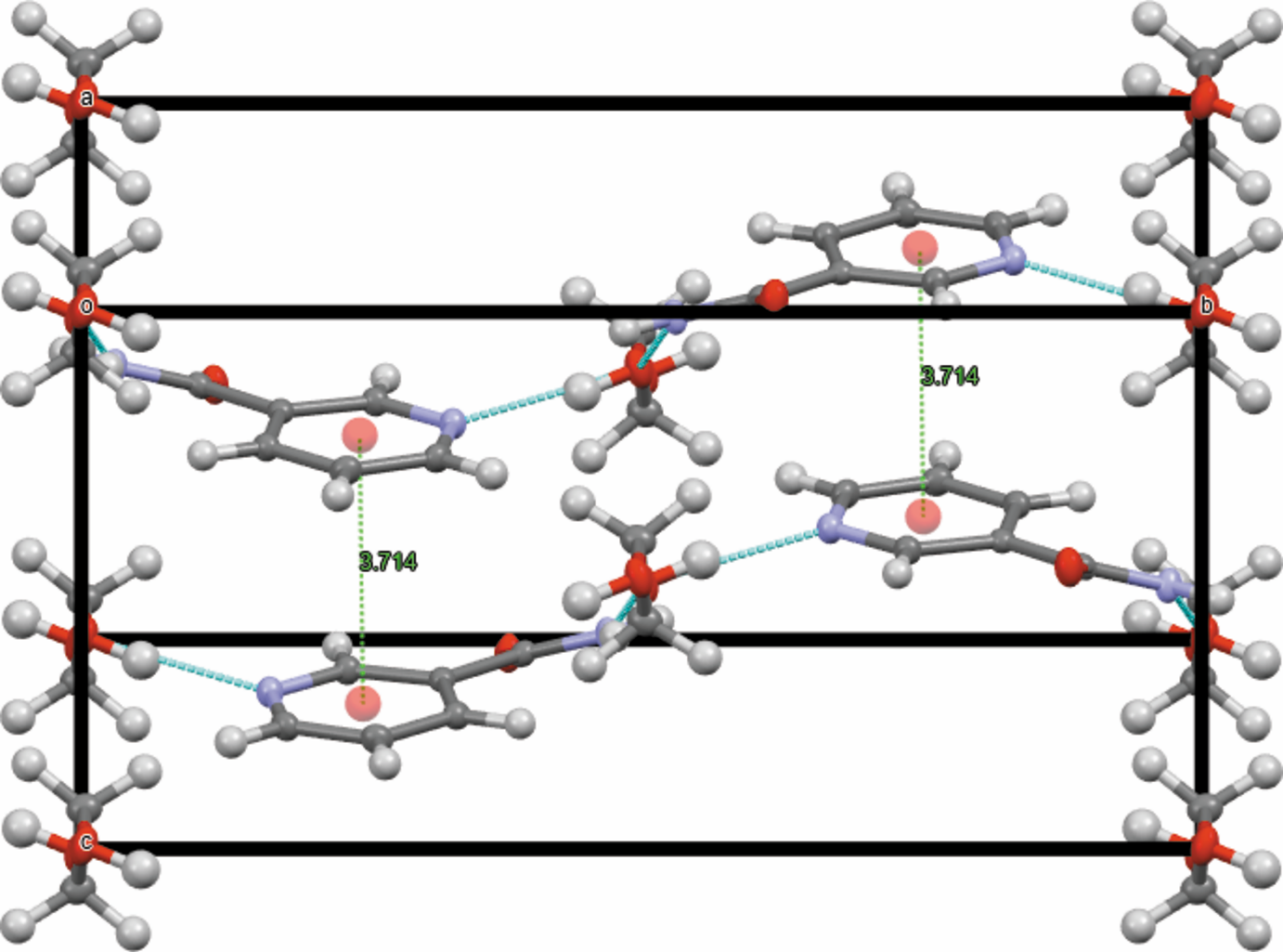
Packing diagram of (**I**) in the unit-cell view from the a* axis. Hydrogen bonds are shown as blue dashed lines, linking neighbor NAM mol­ecules and between EG and NAM mol­ecules. Centroid-to-centroid distances are illustrated as green dashed lines linking each NAM molecule.

In addition, weak off-centered parallel π–π stacking inter­actions are observed between the pyridine rings of adjacent NAM mol­ecules (Martinez & Iverson, 2012 ▸), with a centroid–centroid separation of 3.7138 (6) Å. The angle between the ring planes is 2.57 (5)° with a lateral shift distance of 1.351 Å, confirming the slipped parallel geometry (Figs. 3 ▸ and 4 ▸). The electron-deficient nature of the NAM pyridine ring, induced by its electron-withdrawing groups, further favors the slipped parallel geometry over face-to-face stacking by reducing electrostatic repulsion between adjacent rings. Two C—H⋯O contacts (Table 1 ▸) are identified involving hydrogen atoms H5 and H7 of the pyridine ring with the acceptor oxygen atoms belonging to EG and NAM, respectively.

**Figure 5:**
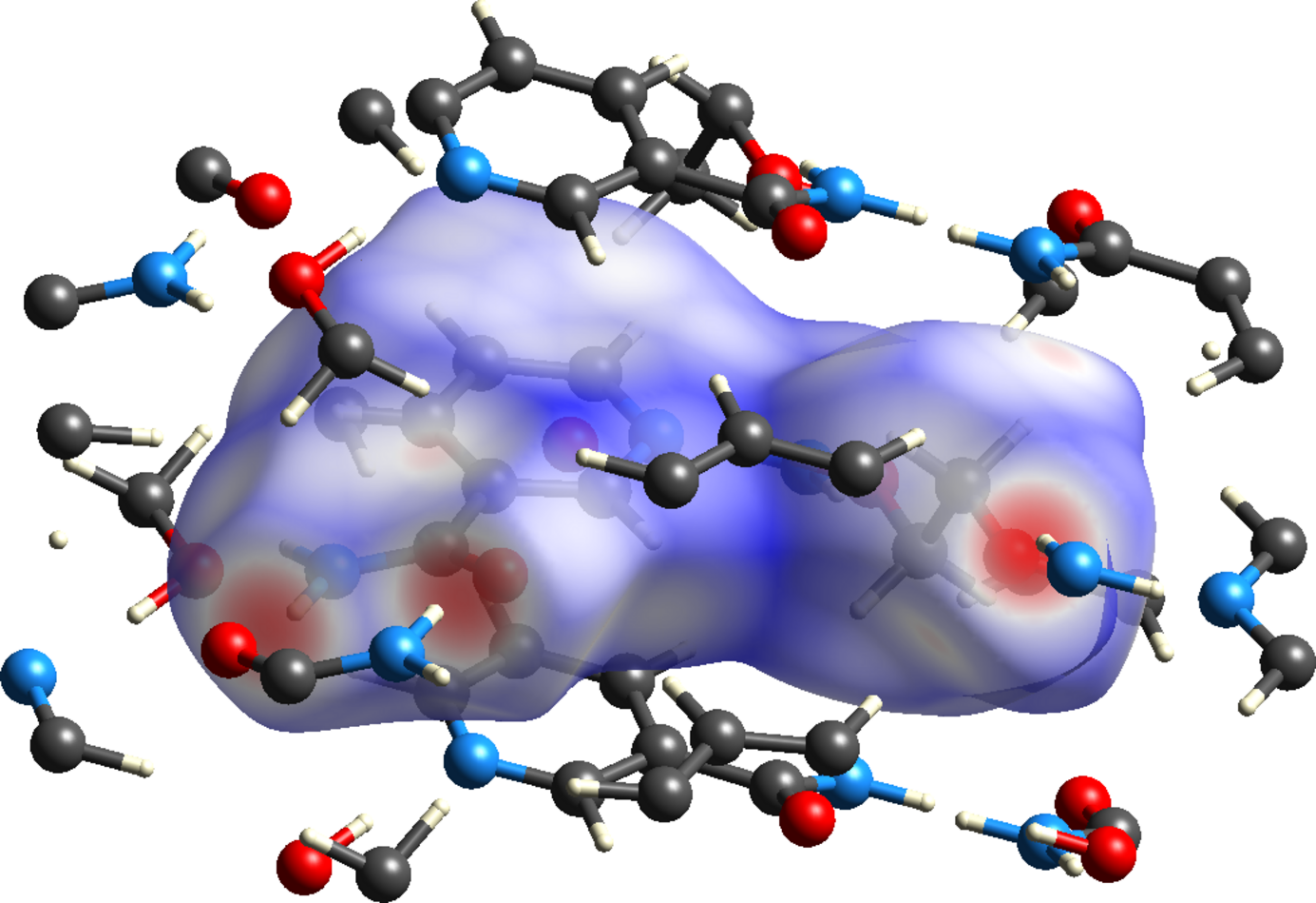
The Hirshfeld surface for (**I**) generated over indicating the significant hydrogen bonds are either N—H⋯O or O—H⋯N inter­actions at the red region.

The overall inter­molecular inter­actions were visualized by Hirshfeld surface analysis, and the fingerprint plots were generated with *Crystal Explorer 21* (Spackman *et al.*, 2021 ▸) and used to identify each contact inside and outside the region of inter­est. The three different colors in the Hirshfeld surface, as depicted in Fig. 5 ▸ indicate each type of inter­action by red regions showing strong and close contact hydrogen bondings, white regions representing contact distances equal to the sum of van der Waals radii, and blue regions indicating contacts that are farther apart than van der Waals radii. From the analysis, the significant hydrogen bonds are N—H⋯O or O—H⋯N inter­actions.

**Figure 6:**
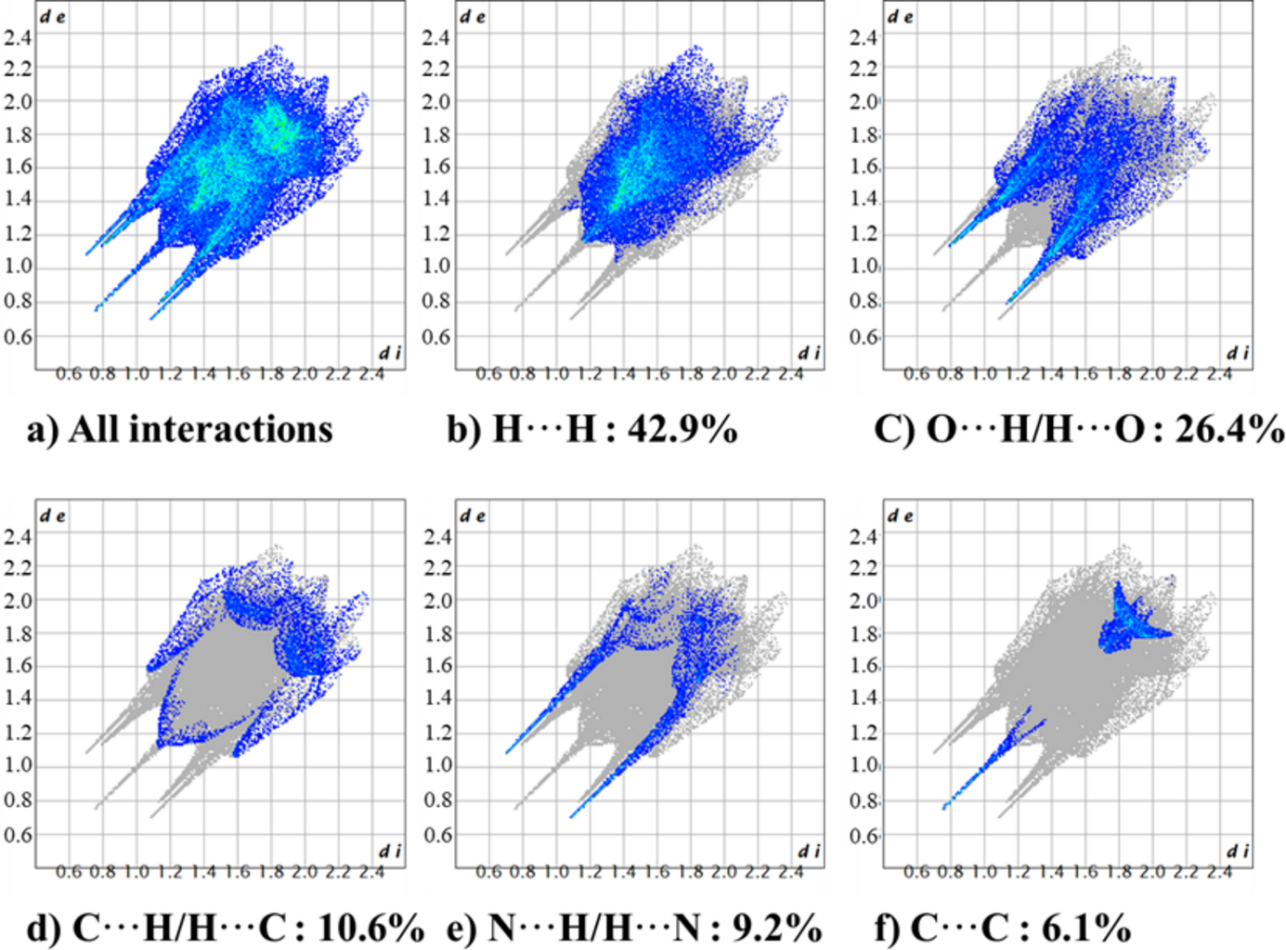
Two-dimensional fingerprint plots for (**I**): (*a*) summary of all inter­actions; specific contributions including (*b*) H⋯H, (*c*) O⋯H/H⋯O, (*d*) C⋯H/H⋯C, (*e*) N⋯H/H⋯N, (*f*) C⋯C, and other minor contributions.

As illustrated in Fig. 6 ▸, the dominant inter­action in the fingerprint plot is H⋯H (42.9%), illustrating weak van der Waals forces. Following, the groups that inter­acted with H atoms, including O⋯H/H⋯O (26.4%), C⋯H/H⋯C (10.6%), and N⋯H/H⋯N (9.2%), expressed as a sharp spike reflecting a shorter distance from the Hirshfeld surface corresponding to close inter­molecular contacts. Finally, the last inter­action, C⋯C (6.1%) illustrates a stacking inter­action between the rings.

## 4. Database survey

Nicotinamide (NAM) is a form of vitamin B3, which was discovered between 1935 and 1937, usually found in food and medication (Sneader, 2005 ▸). A number of NAM crystal structures have been reported in the Cambridge Structural Database (CSD, Version 2025.1.1, last update May 2026; Groom *et al.*, 2016 ▸) with CSD refcodes NICOAM and NICOAM01–NICOAM18. Comparing the conformation of NAM mol­ecule in the structure of (**I**) with these, the bond angles and the dihedral angle of the amide functional group are varied by the position that forms hydrogen bonds and the arrangement of NAM.

Nicotinamide can also form multicomponent crystals with various coformers. One structurally relevant example is the nicotinamide–succinic acid cocrystal (refcode DUZPAQ; Thompson *et al.*, 2010 ▸). Both succinic acid (butane­dioic acid) and ethyl­ene glycol share a –CH_2_–CH_2_– backbone with hydrogen-bond-active groups at each end. However, the proton-donating groups differ: succinic acid carries carb­oxy­lic groups (–COOH) instead of hydroxyl groups (–OH), resulting in stronger hydrogen bond donors and a distinct inter­action geometry. In the case of NAM mol­ecules in DUZPAQ, two hydrogen bonds are still formed at the amide group with a comparable torsion angle, but a dissimilar packing pattern is observed.

## 5. Synthesis and crystallization

The needle-like solvate crystals were obtained by preparing solutions containing EG and NAM. A mixture of NAM (300 mg) in EG (550 µL) was prepared by heating the mixture up to 353 K until the solution was clear, then slowly cooling to 293 K to form the crystal. After cooling, colorless, needle-like crystals of (**I**) could be observed in the solution.

## 6. Refinement

Crystal data, data collection, and structure refinement details are summarized in Table 2 ▸. The O-bound H atom was located in a difference map and its position was freely refined. The other hydrogen atoms were placed at calculated positions using a riding model with N—H = 0.88 and C—H = 0.95–0.99 Å. The constraint *U*_iso_(H) = 1.5 *U*_eq_ (O) and *U*_iso_(H) = 1.2 *U*_eq_ (C, N) was applied in all cases.

## Supplementary Material

Crystal structure: contains datablock(s) global, I. DOI: 10.1107/S2056989026005219/hb8222sup1.cif

CCDC reference: 2477351

Additional supporting information:  crystallographic information; 3D view; checkCIF report

## Figures and Tables

**Figure 4:**
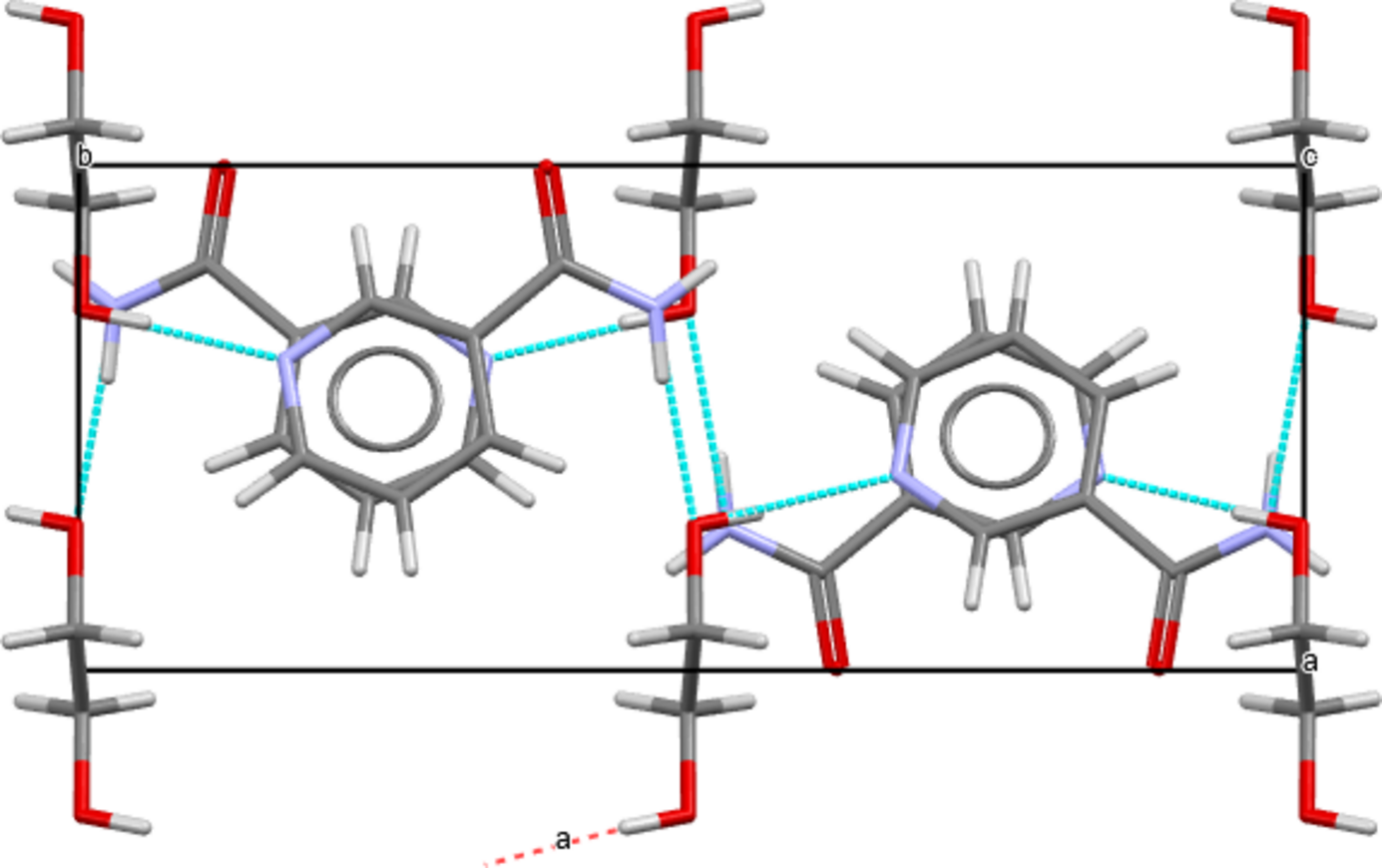
Packing diagram of (**I**) in the unit-cell view from the *c* axis. The structure of NAM in each layer is a mirror image of the other.

**Table 1:** Hydrogen-bond geometry (Å, °)

*D*—H⋯*A*	*D*—H	H⋯*A*	*D*⋯*A*	*D*—H⋯*A*
N1—H1*A*⋯O2^i^	0.88	2.08	2.9372 (13)	165
N1—H1*B*⋯O1^ii^	0.88	2.06	2.8868 (14)	157
O1—H1⋯N2^iii^	0.867 (17)	1.900 (17)	2.7588 (13)	170.7 (13)
C5—H5⋯O1^iv^	0.95	2.62	3.461	147
C7—H7⋯O2^iv^	0.95	2.55	3.323	138

Symmetry codes: (i) 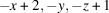; (ii) 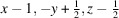; (iii) 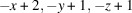; (iv) 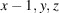.

**Table 2:** Experimental details

Crystal data	
Chemical formula	C_6_H_6_N_2_O·0.5C_2_H_6_O_2_
*M*_r_	153.16
Crystal system, space group	Monoclinic, *P*2_1_/*c*
Temperature (K)	100
*a*, *b*, *c* (Å)	7.0283 (9), 15.5149 (19), 7.4271 (11)
β (°)	114.554 (4)
*V* (Å^3^)	736.64 (17)
*Z*	4
Radiation type	Mo *K*α
μ (mm^−1^)	0.10
Crystal size (mm)	0.5 × 0.4 × 0.2

Data collection	
Diffractometer	D8 Venture diffractometer
Absorption correction	Multi-scan (*SADABS*; Krause *et al.*, 2015 ▸)
*T*_min_, *T*_max_	0.711, 0.745
No. of measured, independent and observed [*I* > 2σ(*I*)] reflections	14232, 1508, 1441
*R*_int_	0.031
(sin θ/λ)_max_ (Å^−1^)	0.626

Refinement	
*R*[*F*^2^ > 2σ(*F*^2^)], *wR*(*F*^2^), *S*	0.033, 0.091, 1.10
No. of reflections	1508
No. of parameters	104
H-atom treatment	H atoms treated by a mixture of independent and constrained refinement
Δρ_max_, Δρ_min_ (e Å^−3^)	0.34, −0.28

Computer programs: *APEX4* and *SAINT* (Bruker, 2016 ▸), *SHELXT2018/2* (Sheldrick, 2015*a* ▸), *SHELXL2018/3* (Sheldrick, 2015*b* ▸), *OLEX2* (Dolomanov *et al.*, 2009 ▸) and *Mercury* (Macrae *et al.*, 2020 ▸).

## supplementary crystallographic information

### Nicotinamide ethylene glycol hemisolvate Crystal data

**Table:**

C_6_H_6_N_2_O·0.5C_2_H_6_O_2_	*F*(000) = 324
*M**_r_* = 153.16	*D*_x_ = 1.381 Mg m^−^^3^
Monoclinic, *P*2_1_/*c*	Mo *K*α radiation, λ = 0.71073 Å
*a* = 7.0283 (9) Å	Cell parameters from 9982 reflections
*b* = 15.5149 (19) Å	θ = 2.6–26.4°
*c* = 7.4271 (11) Å	µ = 0.10 mm^−^^1^
β = 114.554 (4)°	*T* = 100 K
*V* = 736.64 (17) Å^3^	Needle, clear light colourless
*Z* = 4	0.5 × 0.4 × 0.2 mm

### Nicotinamide ethylene glycol hemisolvate Data collection

**Table:**

D8 Venture diffractometer	*R*_int_ = 0.031
Absorption correction: multi-scan (SADABS; Krause *et al.*, 2015)	θ_max_ = 26.4°, θ_min_ = 2.6°
*T*_min_ = 0.711, *T*_max_ = 0.745	*h* = −8→8
14232 measured reflections	*k* = −19→19
1508 independent reflections	*l* = −9→9
1441 reflections with *I* > 2σ(*I*)	

### Nicotinamide ethylene glycol hemisolvate Refinement

**Table:**

Refinement on *F*^2^	Hydrogen site location: mixed
Least-squares matrix: full	H atoms treated by a mixture of independent and constrained refinement
*R*[*F*^2^ > 2σ(*F*^2^)] = 0.033	*w* = 1/[σ^2^(*F*_o_^2^) + (0.0425*P*)^2^ + 0.3016*P*] where *P* = (*F*_o_^2^ + 2*F*_c_^2^)/3
*wR*(*F*^2^) = 0.091	(Δ/σ)_max_ < 0.001
*S* = 1.10	Δρ_max_ = 0.34 e Å^−^^3^
1508 reflections	Δρ_min_ = −0.28 e Å^−^^3^
104 parameters	Extinction correction: SHELXL2018/3 (Sheldrick 2015b), Fc^*^=kFc[1+0.001xFc^2^λ^3^/sin(2θ)]^-1/4^
0 restraints	Extinction coefficient: 0.028 (5)
Primary atom site location: dual	

### Nicotinamide ethylene glycol hemisolvate Special details

**Table:**

Geometry. All esds (except the esd in the dihedral angle between two l.s. planes) are estimated using the full covariance matrix. The cell esds are taken into account individually in the estimation of esds in distances, angles and torsion angles; correlations between esds in cell parameters are only used when they are defined by crystal symmetry. An approximate (isotropic) treatment of cell esds is used for estimating esds involving l.s. planes.

### Nicotinamide ethylene glycol hemisolvate Fractional atomic coordinates and isotropic or equivalent isotropic displacement parameters (Å^2^)

**Table:**

	*x*	*y*	*z*	*U*_iso_*/*U*_eq_	
O1	1.28683 (12)	0.50246 (5)	0.61721 (12)	0.0205 (2)	
O2	0.99610 (11)	0.11854 (5)	0.52304 (12)	0.0203 (2)	
N1	0.71808 (14)	0.02948 (6)	0.38085 (14)	0.0173 (2)	
H1A	0.797694	−0.015059	0.385940	0.021*	
H1B	0.581207	0.023631	0.330179	0.021*	
N2	0.61434 (15)	0.33164 (6)	0.44707 (14)	0.0180 (2)	
C1	1.07962 (17)	0.50292 (8)	0.60563 (17)	0.0207 (3)	
H1C	1.056677	0.556594	0.665922	0.025*	
H1D	1.061588	0.453574	0.681612	0.025*	
H1	1.311 (2)	0.5533 (11)	0.583 (2)	0.031*	
C2	0.80481 (16)	0.10579 (7)	0.44989 (15)	0.0149 (2)	
C3	0.65944 (16)	0.17806 (7)	0.44149 (15)	0.0142 (2)	
C4	0.72994 (17)	0.26247 (7)	0.45120 (16)	0.0162 (2)	
H4	0.867716	0.271516	0.461315	0.019*	
C5	0.42098 (17)	0.31788 (7)	0.43521 (16)	0.0183 (3)	
H5	0.337084	0.366336	0.432227	0.022*	
C6	0.45862 (17)	0.16510 (7)	0.43013 (15)	0.0164 (2)	
H6	0.405209	0.108440	0.424542	0.020*	
C7	0.33818 (17)	0.23615 (7)	0.42710 (17)	0.0185 (3)	
H7	0.200831	0.228994	0.419592	0.022*	

### Nicotinamide ethylene glycol hemisolvate Atomic displacement parameters (Å^2^)

**Table:**

	*U*^11^	*U*^22^	*U*^33^	*U*^12^	*U*^13^	*U*^23^
O1	0.0126 (4)	0.0162 (4)	0.0294 (5)	0.0002 (3)	0.0055 (3)	0.0052 (3)
O2	0.0124 (4)	0.0165 (4)	0.0294 (5)	0.0000 (3)	0.0062 (3)	0.0010 (3)
N1	0.0121 (4)	0.0141 (5)	0.0228 (5)	0.0013 (3)	0.0046 (4)	−0.0011 (3)
N2	0.0187 (5)	0.0152 (5)	0.0204 (5)	0.0000 (3)	0.0086 (4)	−0.0002 (3)
C1	0.0149 (6)	0.0271 (6)	0.0191 (6)	−0.0006 (4)	0.0059 (5)	0.0016 (4)
C2	0.0143 (5)	0.0152 (5)	0.0151 (5)	0.0004 (4)	0.0061 (4)	0.0023 (4)
C3	0.0142 (5)	0.0152 (5)	0.0123 (5)	0.0001 (4)	0.0045 (4)	0.0002 (4)
C4	0.0139 (5)	0.0165 (5)	0.0183 (5)	−0.0005 (4)	0.0070 (4)	0.0002 (4)
C5	0.0187 (5)	0.0177 (5)	0.0191 (5)	0.0037 (4)	0.0085 (4)	−0.0004 (4)
C6	0.0162 (5)	0.0163 (5)	0.0168 (5)	−0.0028 (4)	0.0068 (4)	−0.0010 (4)
C7	0.0148 (5)	0.0221 (6)	0.0203 (6)	−0.0004 (4)	0.0089 (4)	−0.0016 (4)

### Nicotinamide ethylene glycol hemisolvate Geometric parameters (Å, º)

**Table:**

O1—C1	1.4227 (13)	C1—H1D	0.9900
O1—H1	0.868 (18)	C2—C3	1.5007 (14)
O2—C2	1.2388 (13)	C3—C4	1.3914 (14)
N1—H1A	0.8800	C3—C6	1.3931 (15)
N1—H1B	0.8800	C4—H4	0.9500
N1—C2	1.3329 (14)	C5—H5	0.9500
N2—C4	1.3386 (14)	C5—C7	1.3861 (15)
N2—C5	1.3418 (15)	C6—H6	0.9500
C1—C1^i^	1.504 (2)	C6—C7	1.3843 (15)
C1—H1C	0.9900	C7—H7	0.9500
			
C1—O1—H1	107.5 (10)	C4—C3—C6	117.97 (10)
H1A—N1—H1B	120.0	C6—C3—C2	123.35 (9)
C2—N1—H1A	120.0	N2—C4—C3	123.61 (10)
C2—N1—H1B	120.0	N2—C4—H4	118.2
C4—N2—C5	117.55 (9)	C3—C4—H4	118.2
O1—C1—C1^i^	111.22 (12)	N2—C5—H5	118.5
O1—C1—H1C	109.4	N2—C5—C7	122.94 (10)
O1—C1—H1D	109.4	C7—C5—H5	118.5
C1^i^—C1—H1C	109.4	C3—C6—H6	120.5
C1^i^—C1—H1D	109.4	C7—C6—C3	118.90 (10)
H1C—C1—H1D	108.0	C7—C6—H6	120.5
O2—C2—N1	123.37 (10)	C5—C7—H7	120.5
O2—C2—C3	119.67 (9)	C6—C7—C5	119.00 (10)
N1—C2—C3	116.94 (9)	C6—C7—H7	120.5
C4—C3—C2	118.65 (9)		
			
O2—C2—C3—C4	21.70 (15)	C2—C3—C6—C7	178.70 (10)
O2—C2—C3—C6	−156.39 (10)	C3—C6—C7—C5	0.09 (16)
N1—C2—C3—C4	−159.93 (10)	C4—N2—C5—C7	−0.04 (16)
N1—C2—C3—C6	21.98 (15)	C4—C3—C6—C7	0.60 (15)
N2—C5—C7—C6	−0.39 (17)	C5—N2—C4—C3	0.80 (16)
C2—C3—C4—N2	−179.28 (9)	C6—C3—C4—N2	−1.09 (16)

Symmetry code: (i) −*x*+2, −*y*+1, −*z*+1.

### Nicotinamide ethylene glycol hemisolvate Hydrogen-bond geometry (Å, º)

**Table:**

*D*—H···*A*	*D*—H	H···*A*	*D*···*A*	*D*—H···*A*
N1—H1*A*···O2^ii^	0.88	2.08	2.9372 (13)	165
N1—H1*B*···O1^iii^	0.88	2.06	2.8868 (14)	157
O1—H1···N2^i^	0.867 (17)	1.900 (17)	2.7588 (13)	170.7 (13)
C5—H5···O1^iv^	0.95	2.62	3.461	147
C7—H7···O2^iv^	0.95	2.55	3.323	138

Symmetry codes: (i) −*x*+2, −*y*+1, −*z*+1; (ii) −*x*+2, −*y*, −*z*+1; (iii) *x*−1, −*y*+1/2, *z*−1/2; (iv) *x*−1, *y*, *z*.
